# P38 Use of the ‘drug bag method’ to assess household antibiotic use in The Gambia

**DOI:** 10.1093/jacamr/dlaf118.045

**Published:** 2025-07-14

**Authors:** Phoebe Weddle, Stephen Owens, Abigail Lawrence, Karen Forest, Matthew Breckons, Behzad Nadjim

**Affiliations:** Newcastle University Medical School, Newcastle-upon-Tyne, UK; Newcastle University Faculty of Medical Sciences, Newcastle-upon-Tyne, UK; Newcastle University Medical School, Newcastle-upon-Tyne, UK; Clinical Services Department, MRC Unit The Gambia at London School of Hygiene and Tropical Medicine, Banjul, The Gambia; Newcastle University Faculty of Medical Sciences, Newcastle-upon-Tyne, UK; Clinical Services Department, MRC Unit The Gambia at London School of Hygiene and Tropical Medicine, Banjul, The Gambia

## Abstract

**Background:**

Antibiotics are a vital group of medicines designed to treat infections; however, the increase in antimicrobial resistance (AMR) reduces how effective antibiotics are. Consequently, antibiotics have been classified according to how readily they should be used. They are classed as readily available (Access), second line (Watch), and last (Reserve) treatment options. Low-income countries are disproportionally affected by AMR with Western Sub-Saharan Africa having the highest rates of death attributed to AMR in the world. Despite this, there is a paucity of research on antibiotic use in The Gambia. Previously, efforts to estimate antibiotic consumption in low resource settings have relied on simple recall, however, recent studies have implemented the drug bag method. The last estimation of community antibiotic use in The Gambia was recorded in 2012, with 13.6% of households reporting the use antibiotics in the 2 weeks prior to the time of the study.

**Objectives:**

To describe prevalence, patterns and associations of antibiotic use and recognition in The Gambia.

**Methods:**

One hundred and forty households were randomly selected across two regions in The Gambia using Google Earth. With assistance from the ‘drug bag method’ participants were asked to identify antibiotics they recognized or had used. Participants selected from 197 antibiotics. These consisted of every antibiotic type, formulation and brand available in any pharmacy across the study site. A validated WHO survey was adapted and used to collect individual and household demographic information and assess the timings of antibiotic use. Statistical analysis was completed using SPSS.

**Results:**

Amongst the 140 households sampled, point prevalence of antibiotic use (including topical antibiotics) was 21.4% of households (95% CI: 14.6%–28.2%). Without topical antibiotics, point prevalence was 13.6% (95% CI:7.9%–19.2%). Over 70% of the households recognized at least one Watch antibiotic. Households larger than the national average or households with children were more likely to recognize a Watch antibiotic in analysis.

**Conclusions:**

The overall point prevalence is higher than previously estimated. This is reflective of the inclusion of topical antibiotics, previously excluded in literature. The point prevalence excluding topical antibiotics was consistent with the 2012 estimation, however, these studies were performed during different weather seasons and therefore may not be directly comparable. Increased exposure to Watch antibiotics amongst households with children may reflect global treatment trends. The reasons for these trends are not fully understood. Further research needs to be completed to assess the appropriateness of antibiotic use in The Gambia and long-term monitoring of antibiotic use needs to be established.

